# Load-Dependent Prefrontal Cortex Activation Assessed by Continuous-Wave Near-Infrared Spectroscopy during Two Executive Tasks with Three Cognitive Loads in Young Adults

**DOI:** 10.3390/brainsci12111462

**Published:** 2022-10-28

**Authors:** Nounagnon Frutueux Agbangla, Michel Audiffren, Jean Pylouster, Cédric T. Albinet

**Affiliations:** 1 Unité de Recherche Pluridisciplinaire Sport Santé Société (URePSSS), Université d’Artois, Université de Lille, Université du Littoral Côte d’Opale, ULR 7369, 62800 Liévin, France; 2 Centre de Recherches sur la Cognition et l’Apprentissage, Université de Poitiers, CNRS, Maison des Sciences de l’Homme et de la Société, 5 Rue Théodore Lefebvre TSA 21103, CEDEX 9, 86073 Poitiers, France; 3 Laboratoire Sciences de la Cognition, Technologie, Ergonomie (SCoTE-EA7420), Université de Toulouse, INU Champollion, 81012 Albi, France

**Keywords:** fNIRS, cerebral oxygenation, working memory, inhibition, executive functions, cognitive load, n-back, random number generation

## Abstract

The present study examined the evolution of the behavioral performance, subjectively perceived difficulty, and hemodynamic activity of the prefrontal cortex as a function of cognitive load during two different cognitive tasks tapping executive functions. Additionally, it investigated the relationships between these behavioral, subjective, and neuroimaging data. Nineteen right-handed young adults (18–22 years) were scanned using continuous-wave functional near-infrared spectroscopy during the performance of n-back and random number generation tasks in three cognitive load conditions. Four emitter and four receptor optodes were fixed bilaterally over the ventrolateral and dorsolateral prefrontal cortices to record the hemodynamic changes. A self-reported scale measured the perceived difficulty. The findings of this study showed that an increasing cognitive load deteriorated the behavioral performance and increased the perceived difficulty. The hemodynamic activity increased parametrically for the three cognitive loads of the random number generation task and in a two-back and three-back compared to a one-back condition. In addition, the hemodynamic activity was specifically greater in the ventrolateral prefrontal cortex than in the dorsolateral prefrontal cortex for both cognitive tasks (random number generation and n-back tasks). Finally, the results highlighted some links between cerebral oxygenation and the behavioral performance, but not the subjectively perceived difficulty. Our results suggest that cognitive load affects the executive performance and perceived difficulty and that fNIRS can be used to specify the prefrontal cortex’s implications for executive tasks involving inhibition and working memory updating.

## 1. Introduction

Executive functions (EFs) are high-level processes that facilitate an adaptation to unusual situations when the highly practiced cognitive abilities or behavior do not suffice [1]. The literature describes the main EFs as controlled inhibition, working memory updating, and cognitive flexibility [2]. Concerning the controlled inhibition and working memory updating, numerous studies have developed and used different experimental tasks to examine the involvement of these core EFs in behavioral paradigms. The most used tasks to examine controlled inhibition are Stroop-like tasks, go/no-go tasks, flanker tasks, or stop-signal tasks [3,4]. The most used tasks to assess updating require being able to dynamically monitor and update the verbal or spatial information in the working memory, such as in n-back tasks [5] or running span tasks [6]. Among all these tasks, and directly in relation to the present research, some studies have explored the neural substrates that underlie these EFs using the n-back and random number generation (RNG) tasks that solicit working memory updating [7,8,9] and controlled inhibition [10,11,12], respectively. During an n-back task, one must decide whether the presented stimulus matches the one presented in the n-trials before in a sequence of multiple stimuli, which requires continuously updated the working memory content. During an RNG task, one must usually generate a string of specified numbers (e.g., from one to nine) at a constant rate as randomly as possible, which requires monitoring the working memory and inhibiting overlearned schemas (i.e., counting). 

Regarding working memory updating, as measured by the n-back task, functional magnetic resonance imaging (fMRI) studies have shown that the bilateral and medial posterior parietal cortex, bilateral premotor cortex, dorsal cingulate/medial premotor cortex, bilateral rostral prefrontal cortex, bilateral dorsolateral prefrontal cortex (DLPFC), bilateral mid ventrolateral prefrontal cortex (VLPFC), bilateral precuneus, and left anterior insula and bilateral thalamus regions are consistently activated [5,9,13]. Other studies that have used functional near-infrared spectroscopy (fNIRS) highlighted the role of the prefrontal cortex (PFC) during the n-back task [8,14]. Indeed, the hemodynamic activity of the PFC was shown to be bilateral [7], but with a strong activation of the left hemisphere, particularly for the verbal version of the n-back task [14]. In addition, other studies have shown that the hemodynamic activity of the PFC increases substantially with an increasing working memory load [15,16,17]. According to Vermeij and collaborators, the hemodynamic activity of the right PFC in young participants was slightly dominant at low levels of working-memory load (0-back, 1-back), while it became more bilateralized at a higher cognitive load (e.g., two-back) [17]. On the contrary, Saikia and collaborators also showed an increased bilateral PFC activation as a function of an increasing cognitive load (from one-back to three-back) and reported a slightly greater overall activation over the left PFC [16]. Further studies should address these discrepancies, acknowledging that these two fNIRS studies did not differentiate between the DLPFC and VLPFC. 

In contrast to the n-back task, few studies have examined the neural substrates of the controlled inhibition performance using the RNG task. Indeed, Jahanshahi and collaborators used a positron emission tomography (PET) scan to show that relative to a control counting task, RNG was associated with significant activations in the left DLPFC, the anterior cingulate, the bilateral superior parietal cortex, the right inferior frontal cortex, and the bilateral cerebellar hemispheres. However, when RNG increased (from 0.33 Hz to 2 Hz), a decrease was observed in the performance, along with a decrease in the regional cerebral blood flow (rCBF) in the left and right DLPFC and the right superior parietal cortex. Moreover, they reported a specific negative correlation between the activation of the left DLPFC and RNG behavioral performance [12]. In the same vein, Daniels and collaborators used fMRI and observed similar brain activations as Jahanshahi and collaborators during RNG with a generation rate of 1 Hz. On the contrary, when the generation rate increased to 2 Hz (increasing the task difficulty), the activation of all these same brain areas declined along with the performance [11]. Based on these two studies, it can be hypothesized that in difficult high-rate conditions, cognitive processes responsible for the suppression of habitual responses (inhibition) are less active in maintaining the high generation frequency; thus, when the capacity limits are exceeded, a decrease in the PFC activity is coupled with a performance deterioration.

One original study, which is of particular importance for the present research, examined the hemodynamic activity in the DLPFC and VLPFC during the two abovementioned tasks using the same protocol with the same individuals [18]. Considering that brain activity can vary according to the executive processes required to perform each task, the authors explored the degree of prefrontal activations in both tasks using fNIRS. The results of this study have supported a dominant hemodynamic activity both in the left and right VLPFC during the n-back task. During the RNG task, the hemodynamic activity was much more diffuse in the entire PFC. In their study, Hoshi and collaborators used three cognitive loads for the n-back task but only one for the RNG task [18]. Even though this study allowed for determining the specific involvement of the PFC in both tasks, it did not allow for simultaneously exploring the effect of cognitive load on both tasks.

The effect of the cognitive load on the hemodynamic activity has been widely demonstrated in the n-back task using fNIRS [7,8,15,16,17,19,20] and fMRI [21], and in others working memory tasks, using fMRI [22,23]. For the most part, these studies relied on neurocognitive models describing the augmented hemodynamic activity in supplementary brain areas other than the preliminary specialized ones, as a function of an increasing cognitive load e.g., [24,25,26]. These theoretical models were developed to explain the normal cognitive and cerebral aging and whether this augmented brain activity is compensatory or not is still under debate [27,28,29]. In contrast, very few studies have tested the effect of cognitive load using the RNG task. To our knowledge, only the study of Albinet and collaborators showed this effect during the RNG tasks, but only in older adults [10]. This study, using only two difficulty levels, showed that the DLPFC activity increased bilaterally when the generation rate increased from 0.66 to 1 Hz. Moreover, the cognitive performance was significantly and positively correlated with the left and right DLPFC activity, but only at the lowest 0.66 Hz rate condition. The first objective of the present study was thus to examine the simultaneous evolution of the cognitive performance and the PFC hemodynamic activity as a function of three levels of cognitive load in the n-back task and the RNG task in the same pool of young adults. This will allow us to study the possible distinct patterns of cerebral activity during the performance of these two executive tasks and explore whether their respective evolution as a function of task difficulty is equivalent. 

One important point that should be addressed concerns the fact that the cognitive load, manipulated objectively and externally by an increasing task difficulty (e.g., increasing the number of items to be updated in the working memory or increasing the rate of random generation or the number of information to be inhibited), may not directly match the individual mental workload, that is the perceived difficulty of the task experimented by an individual and the cognitive effort put in it. Neuroimaging techniques can help better understand the mental workload [30], and several fNIRS studies have reported a dissociation between the cognitive performance and brain activity [19,31,32]. Based on the neural efficiency hypothesis [33], some people would need to use a substantial number of cerebral resources for a given cognitive performance, while others would perform the same with less effort and thus less cerebral activity. These differences can be linked to individual differences, such as age, gender, expertise, and task difficulty [34]. It is thus important to monitor, for any prior defined cognitive load, the actual subjective perceived difficulty by an individual. This can be done using a validated questionnaire [35] or a self-reported scale [36]. A second objective of the present study was thus to examine whether the variations in the PFC activity relate more to the cognitive performance or the subjectively perceived difficulty.

Based on previous works, we expected that: (1) an increased cognitive load will lead to an increased subjective perceived difficulty and impaired cognitive performance; (2) an increased cognitive load will result in a bilateral increase in the hemodynamic activity, particularly in the VLPFC for the n-back task, but more diffuse in both the VLPFC and DLPFC for the RNG task; and (3) significant correlations between the behavioral data, subjectively perceived difficulty, and hemodynamic activity until the capacity limits are exceeded, in which case these relationships should be disrupted.

## 2. Materials and Methods

### 2.1. Participants 

The sample size estimation was based on the reviewed literature reported in Section 1, which examined the prefrontal hemodynamic activity using fNIRS during the performance of n-back or RNG tasks [7,8,16,17,18,19,20]. Nineteen right-handed young adults participated in the study. Among these, there were 17 men and 2 women. Their mean age was 19.7 ± 1 years and they were in good cardiorespiratory fitness (54.8 ± 7.2 mL/Kg/min) with a high level of education (14 ± 0 years). None of them suffered from cardiovascular or neurological pathology. All participants completed and signed the informed written consent form before participating in the study, which was approved by the local ethics committee (CERNI Tours-Poitiers n° 2015-04-02) following the ethical standards laid down in the Declaration of Helsinki.

### 2.2. Cognitive Assessment

#### 2.2.1. n-Back Task

The n-back task is usually used to investigate the updating of the working memory [37]. In this task [15], participants must decide as accurately and quickly as possible whether each letter matches the one presented in the n-trials before in the sequence (n є {1,2,3}). Each participant performed three conditions of the n-back task (1, 2, 3-back) and a control task (0-back), in which they had to decide whether the presented letter was an “X”. Each condition contained 42 stimuli with 14 targets and 28 non-targets and lasted for 150 s. In each condition, the stimuli presentation was controlled by E-Prime^®^ software 2.0 (Psychology Software Tools, Sharpsburg, PA, USA), which recorded the response times (RT) and response accuracy. Before the appearance of each stimulus, a black cross appeared on the screen for 500 ms. Then, the stimulus was displayed, and the participants had 3000 ms to respond. The participants responded by pressing one of two keys (yes/no) of the Serial Response Box^TM^ (Psychology Software Tools, Sharpsburg, PA, USA) with their right or left index fingers. We calculated an accuracy score (score A’) using the following formula: 0.5 + ((hit rate − false alarm rate) × (1 + hit rate − false alarm rate))/(4 × hit rate × (1-false alarm rate)) [38]. The higher the A’ score, the better the cognitive performance.

#### 2.2.2. Random Number Generation Task

RNG is a cognitive task involving executive control, particularly of the inhibition function [39,40]. This task requires the participant to say aloud a number from 1 to 9 at a constant rate, paced by a metronome, such that the string of numbers is in an order that is as random as possible [40]. The manipulation of the three cognitive loads of this task was determined based on the previous work of Towse and Valentine [41]. At the first cognitive load (RNG1), the participants said numbers between 1 and 9 at a rate of 1 digit/1.5 s (0.66 Hz). During the two other cognitive loads (RNG2 and RNG3), the participants said numbers between 1–0 and 1–11, avoiding the number 4 in the second cognitive load and numbers 3 and 6 in the third cognitive load, at the same constant rate of 1 digit/1.5 s (0.66 Hz). These two later conditions were imposed on the participants to inhibit saying these specific numbers aloud. In addition to these three cognitive loads, participants also performed a counting task as a control condition (CNT), which involved repeatedly saying aloud the numbers in the order of 1–9 at the same rate [10]. Each participant’s response was recorded using a Dictaphone (Philips voice tracer digital recorder LFH0652) and transcribed by the experimenter. The inhibition performance was measured for each cognitive load by the adjacency score using the RGcalc software [40]. The adjacency is the ratio between the adjacent digits (ascending or descending, i.e., 4–5, 3–2) and the participants’ generated total response pairs. The range varied from 0 to 100%, with a greater score indicating a poorer executive performance and a tendency to count in ascending or descending order [10]. In addition to the adjacency score, we estimated the success rate (i.e., the percentage of responses said aloud following the tempo). Each RNG task condition lasted 150 s.

### 2.3. Apparatus

Relative concentration changes in oxyhemoglobin [O_2_Hb] and deoxyhemoglobin [HHb] were collected using a two-wavelength (857 and 764 nm) continuous-wave spectrometer (Oxymon MKIII, Artinis Medical Systems ^BV^, Zetten, The Netherlands). This tool allows for calculating the relative changes in [O_2_Hb] and [HHb] using the modified Beer–Lambert law [42]. The differential pathlength factor (DPF) was determined using the formula DPF (λ = 807 nm, A) = 4.99 + 0.067 × (age 0.814) [43]. In our study, the DPF ranged from 5.69 to 5.81. Data were collected at a sampling rate of 10 Hz. To measure the relative changes in [O_2_Hb] and [HHb], we used four channels (two transmitter and two receiver optodes, see [20]), which were fixed on the right and left PFC (BA 9/46 and BA 47/45/44) using the international 10/20 system [44] with an inter-optode distance of 3.5 cm [45]. During the experiment, the participants were asked to avoid abrupt head movements, frowning, and tightening their jaws to minimize the noise in the hemodynamic signals.

### 2.4. fNIRS Data Analysis

**Figure 1:**
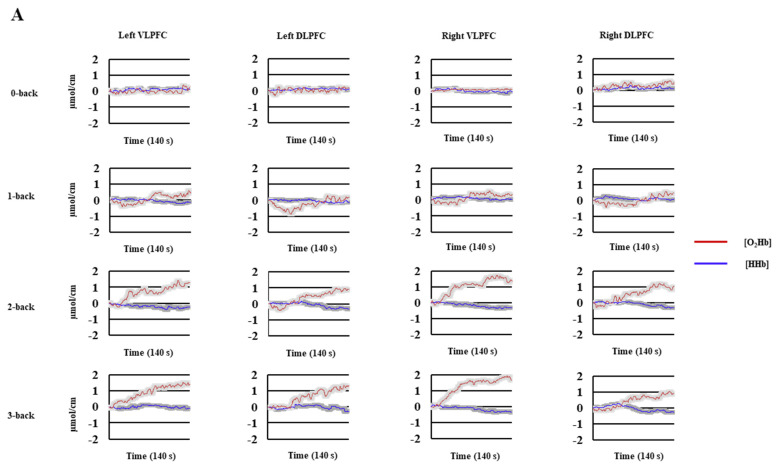

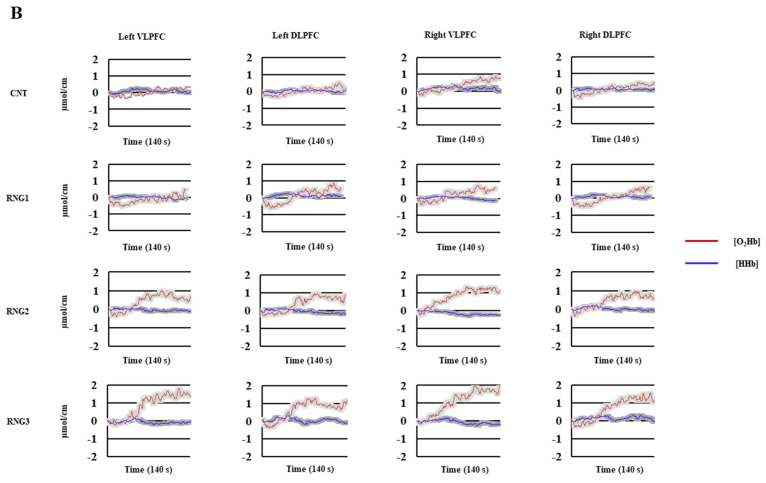
Mean hemodynamic activity as a function of cognitive load, hemisphere, and channel. (**A**) = n-back; (**B**) = RNG (random number generation). The colored frames on the curves correspond to the standard error of the mean; VLPFC = ventrolateral prefrontal cortex; DLPFC = dorsolateral prefrontal cortex.

We used standard procedures that have already been adopted in data processing previously [15,17]. First, the raw data were converted into the optical density, which was then converted into ΔO_2_Hb and ΔHHb using the modified Beer–Lambert Law. A moving Gaussian window of 1 s was applied to the hemodynamic signals to filter the noise of the heartbeat frequency. Matlab’s movement artifact reduction algorithm (MARA) [46] was used to remove the potential motion artifacts. After the filtering process, a bias removal was made 10 s after the beginning of each experimental condition [15,17]. The grand average of the mean hemodynamic activity during each executive task condition is summarized in Figure 1. Then, the area under the curve (AUC) was calculated for [O_2_Hb] and [HHb] utilizing the approximate integration method [47,48] for each experimental condition (control conditions: 0-back and CNT; cognitive load: 1, 2, 3-back and RNG1, RNG2, RNG3). Finally, we subtracted the AUC of the control conditions from each cognitive load of both tasks [18] to determine the specific patterns of the cerebral activation.

### 2.5. Procedure

The participants were seated in front of a computer screen in a quiet, dimly lit room. They completed the Edinburgh Handedness Inventory [49] to ascertain that they were all right-handed. Afterward, the experimenter installed the fNIRS optodes on the forehead of the participants. Subsequently, they performed the n-back or RNG tasks in a counterbalanced order. During the n-back task, the participants performed the three conditions of the n-back (1, 2, 3-back), which were counterbalanced using a modified Latin square [50]. Before and after these three conditions, they performed the 0-back control condition. Only the second realization of the control condition was considered for statistical analyses because the first realization was used as a mock trial.

**Figure 2:**
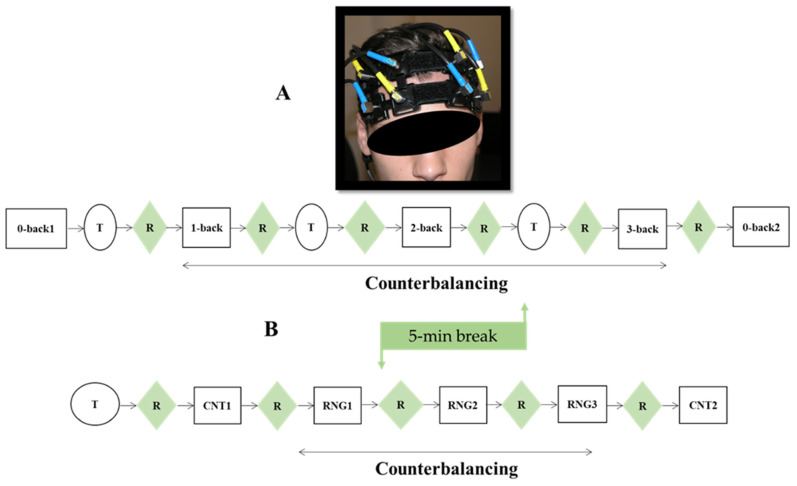
(**A**) Picture of the optode configuration setup on the forehead of one participant. (**B**) Schematic representation of the experimental course. R = 90-s pause; T = training; n-back counterbalancing = 1, 2, 3-back–1, 3, 2-back–2, 1, 3-back–2, 3, 1-back–3, 1, 2-back–3, 2, 1-back; RNG counterbalancing = RNG1, 2, 3–RNG1, 3, 2–RNG2, 1, 3–RNG2, 3, 1–RNG3, 1, 2–RNG3, 2, 1.

Participants took part in a training phase before performing each task condition. A resting period of 90s followed each condition. The RNG task was performed following the same procedure as the n-back task. At the end of the first task, a 5 min break was given to the participants before they performed the second task. The hemodynamic data were continuously recorded during both cognitive tasks. To evaluate the subjectively perceived difficulty of each cognitive load, the participants completed a validated 15-point self-report scale [36] at the end of each condition. Based on Borg’s work and his RPE scale [51], this scale was assessed on a scale from “extremely easy” (1) to “extremely difficult” (15), with higher scores indicating greater subjective perceptions of the task difficulty. The whole experimental protocol lasted 1 h and 30 min. A picture representing the positioning of the pairs of fNIRS optodes and a schematic of the whole procedure are presented in Figure 2.

### 2.6. Statistical Analysis

All statistical analyzes were performed using Statistica software version 12 (StatSoft, France). The normality and homogeneity of the data were assessed using Kolmogorov–Smirnov and Levene tests [52]. Arc sinus transformations were performed on the success rate of the RNG and score A’ of the n-back, which were not normally distributed. The subjectively perceived difficulty of the n-back and RNG was analyzed with a 2 (task: n-back vs. RNG) × 4 (cognitive load: 0, 1, 2, 3-back vs. CNT, RNG1, RNG2, RNG3) repeated-measures MANOVA with the cognitive load and task as the within-subject factors. For the behavioral performance of the n-back, we conducted a repeated-measures MANOVA on RT (0 vs. 1 vs. 2 vs. 3-back) of the correct responses and score A’ with the cognitive load as a within-subject factor. The same repeated-measures MANOVA was used to analyze the behavioral performance of the RNG task (adjacency and success rate (RNG1 vs. RNG2 vs. RNG3)). For [O_2_Hb] and [HHb], a 2 (Task: n-back vs. RNG) × 2 (Hemisphere: left vs. right) × 2 (channel: DLPFC vs. VLPFC) × 3 (Activation: 1 vs. 2 vs. 3-back and RNG1 vs. RNG2 vs. RNG3) repeated-measure MANOVA was performed to compare the activation of both tasks, with the hemisphere, channel, and cognitive load as the within-subject factors. Afterward, for each task separately, a 2 (Hemisphere: left vs. right) × 2 (channel: DLPFC vs. VLPFC) × 3 (Activation: 1 vs. 2 vs. 3-back and RNG1 vs. RNG2 vs. RNG3) repeated-measures MANOVA was performed, with the hemisphere, channel, and cognitive load as the within-subject factors. Repeated contrasts were used to compare the subjective perceptions of the difficulty, behavioral performance, and prefrontal hemodynamic activity of the different cognitive loads. Finally, for each task, the relationships between the behavioral performance and subjective perceptions of the difficulty and [O_2_Hb] changes were examined using the correlation analyses for each cognitive load. All data were expressed as the mean ± SD. The level of significance was set at *p* < 0.05.

## 3. Results

### 3.1. Subjective Perceived Difficulty

**Figure 3:**
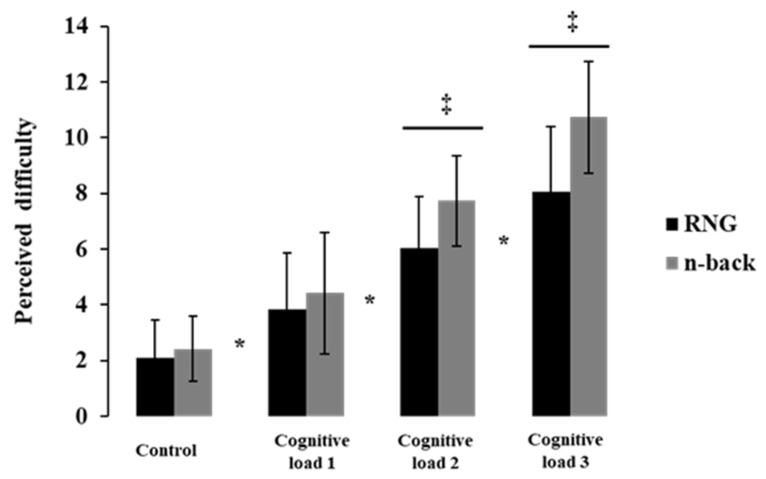
The perceived difficulty as a function of cognitive load and task. * *p* < 0.05 significant difference between control (CNT or 0-back) and others cognitive load (cognitive load 1: RNG1 or 1-back; cognitive load 2: RNG2 or 2-back; cognitive load3: RNG3 or 3-back); ‡ *p* < 0.05 significant difference between the tasks (RNG vs. n-back) during cognitive load 2 and cognitive load 3; error bars indicate standard error.

The MANOVA revealed the significant main effects of the task [F (1,17) = 20.3; *p* < 0.05; Wilks’ lambda = 0.45] and cognitive load [F (3,15) = 53.55; *p* < 0.05; Wilks’ lambda = 0.08] and the significant task × cognitive load interaction [F (3, 15) = 7.09; *p* < 0.05; Wilks’ lambda = 0.41]. The planned contrasts indicated significant differences between the two-back (7.74 ± 1.63) and RNG2 (6.05 ± 1.84) and between the three-back (10.74 ± 2) and RNG3 (8.05 ± 2.34) in the subjectively perceived difficulty (*p* < 0.05; see Figure 3). These results imply that for the second cognitive load, the subjectively perceived difficulty of the n-back was significantly greater than the subjectively perceived difficulty of the RNG. Individual data are presented in the Supplementary Materials.

### 3.2. Behavioral Performances

#### 3.2.1. n-Back

The MANOVA showed the main effect of the cognitive load on RT [F (3, 16) = 12.20; *p* < 0.05 Wilks’ lambda = 0.3], indicating that the RT increased significantly during the two-back (*p* < 0.05) and the three-back (*p* < 0.05) conditions compared to the 0-back and the 1-back. In addition, the two-back and three-back conditions differed significantly (*p* = 0.05). Conversely, no significant difference emerged between the 0-back and 1-back conditions (*p* = 0.56). Regarding the score A´, the MANOVA revealed the main effect of the cognitive load [F (3, 16) = 72.48; *p* < 0.05; Wilks’ lambda = 0.06]. The planned contrasts indicated a significant decrease in the score A’ during the two-back (*p* < 0.05) and three-back (*p* < 0.05) conditions compared to the zero-back and one-back. In addition, between the two-back and three-back conditions, the decrease in the score A’ was also significant (*p* < 0.001). In contrast, the zero-back and one-back conditions did not differ significantly in the score A’ (see Table 1). As a reminder, a decrease in the score A’ reflects a deterioration of the cognitive performance. Individual data are presented in the Supplementary Materials.

#### 3.2.2. RNG

The MANOVA showed the main effect of the cognitive load on the adjacency [F (2, 17) = 4.13; *p* = 0.034; Wilks’ lambda = 0.67] and on the success rate [F (2, 17) = 18.15; *p* < 0.05; Wilks’ lambda = 0.31]. The planned contrasts indicated a significant increase in the adjacency during the RNG3 (*p* = 0.04) compared to RNG1. However, between the RNG2 and RNG3, there was no significant difference (*p* = 0.07), as well as between the RNG2 and RNG1 (*p* = 0.55). As a reminder, an increase in the adjacency reflects a deterioration of the cognitive performance. In contrast, the success rate decreased during the RNG3 compared to RNG1 (*p* < 0.05) and RNG2 (*p* = 0.001) (see Table 1). Individual data are presented in the Supplementary Materials.

### 3.3. fNIRS Data

During the n-back and RNG tasks, one (i.e., 5%) and three participants (i.e., 15%) were excluded, respectively, because of too many corrupted signals. Therefore, 18 and 16 participants were included in the fNIRS data analyses of the n-back and RNG, respectively. Overall, most of the hemodynamic data (86.3%) showed a classic activation pattern, with an increase in [O_2_Hb] and a concomitant slight decrease in [HHb].

The MANOVA, examining the effects of the task, hemisphere, channel, and cognitive load on the AUC of [O_2_Hb] and [HHb], showed no significant effect of the task (all *ps* > 0.48). The analysis showed significant main effects of the channel [F (1, 15) = 39.11; *p* < 0.05; Wilks’ lambda = 0.27] and cognitive load [F (2, 14) = 15.28; *p* < 0.05; Wilks’ lambda = 0.31] on [O_2_Hb]. Concerning the AUC of [HHb], the MANOVA also revealed the main effects of the channel [F (1,15) = 4.54; *p* < 0.05; Wilks’ lambda = 0.76] and cognitive load [F (2,14) = 7.3; *p* < 0.05; Wilks’ lambda = 0.48]. Globally, as detailed below, the activation increased as a function of the cognitive load for both tasks, with a greater activation of the VLPFC than the DLPFC. 

#### 3.3.1. n-Back

**Figure 4:**
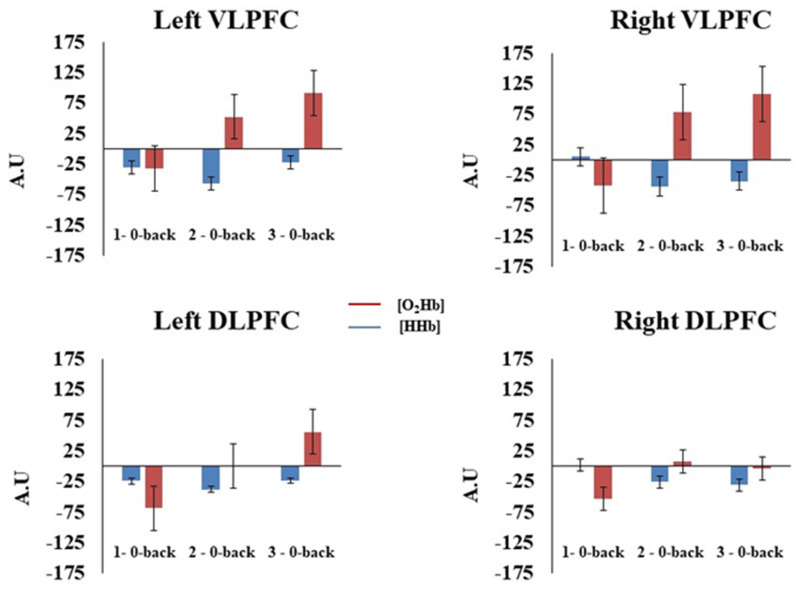
Hemodynamic concentration changes in the prefrontal cortex as a function of cognitive load, hemisphere, and channel during n-back. Bars represent standard error. 1–0-back = Activation 1; 2–0-back = Activation 2; 1–0-back = Activation 3. VLPFC = ventrolateral prefrontal cortex; DLPFC = dorsolateral prefrontal cortex.

The MANOVA showed the main effect of the channel [F (1, 17) = 11.53; *p* < 0.05; Wilks’ lambda = 0.59], indicating a greater AUC of [O_2_Hb] in the VLPFC as compared to the DLPFC. The analysis also revealed that the mean AUC of [O_2_Hb] increased as a function of the cognitive load [F (2, 16) = 6.93; *p* < 0.05; Wilks’ lambda = 0.53]. The planned contrasts indicated a significant increase in [O_2_Hb] during the two-back (*p* < 0.05) and three-back (*p* < 0.05) compared to the one-back, but no significant difference emerged between the two-back and three-back conditions (*p* = 0.33). Concerning the AUC of [HHb], the MANOVA showed a significant hemisphere × cognitive load interaction [F (2, 16) = 5.71; *p* < 0.05; Wilks’ lambda = 0.58]. The planned contrasts indicated that the AUC of [HHb] was significantly more negative in the left hemisphere compared to the right hemisphere during the one-back (*p* = 0.01) condition. However, this difference was not significant during the two-back (*p* = 0.67) and three-back (*p* = 0.86) conditions (see Figure 4).

#### 3.3.2. RNG

**Figure 5:**
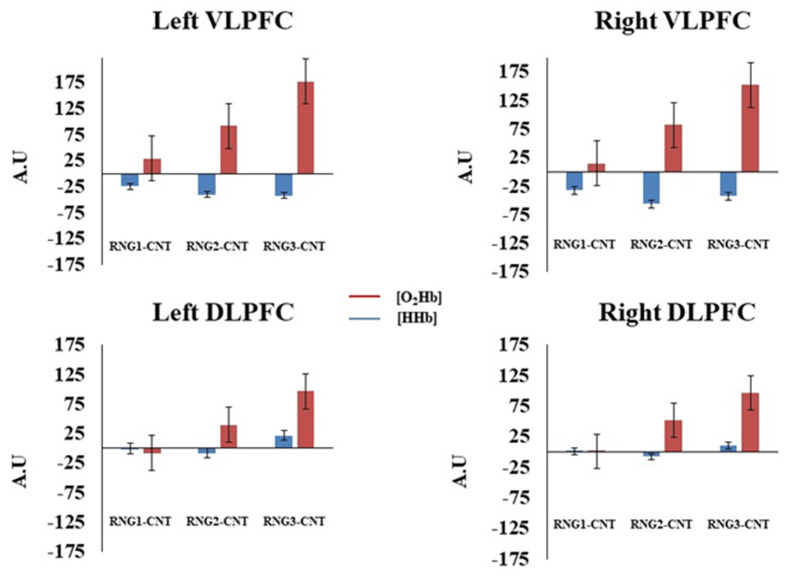
Hemodynamic concentration changes in the prefrontal cortex as a function of cognitive load, hemisphere, and channel during random number generation. Bars represent standard error. RNG1-CNT = activation 1; RNG2-CNT = activation 2; RNG3-CNT = activation 3; VLPFC = ventrolateral prefrontal cortex; DLPFC = dorsolateral prefrontal cortex.

The MANOVA revealed the main effects of the channel [F (1, 15) = 5.63; *p* < 0.05; Wilks’ lambda = 0.72] and cognitive load [F (2, 14) = 9.68; *p* < 0.05; Wilks’ lambda = 0.41]. The AUC of [O_2_Hb] was significantly greater in the VLPFC compared to DLPFC. Furthermore, the planned contrasts indicated that the AUC of [O_2_Hb] was significant greater during the RNG2 compared to RNG1 (*p* < 0.05) and greater during the RNG3 compared to RNG2 (*p* < 0.05). The MANOVA performed on the AUC of [HHb] showed the main effect of the channel [F (1, 15) = 11.80; *p* < 0.05; Wilks’ lambda = 0.56]. This result indicated that the AUC of [HHb] was significantly more negative in the VLPFC compared to DLPFC (see Figure 5).

### 3.4. Relationships between Behavioral Performance, Perceived Difficulty, and [O_2_Hb] Changes

#### 3.4.1. n-Back

Score A´ correlated significantly negatively with the AUC value of [O_2_Hb] of the right DLPFC during the one-back (*r* = −0.52; *p* = 0.027) and during the three-back (*r* = −0.55; *p* = 0.019) but not during the two-back (*r* = −0.30; *p* = 0.221) conditions. These results indicated that an improved behavioral performance was associated with less activation in the right DLPFC during the one-back and three-back tasks. The RT values did not correlate significantly with any hemodynamic value. Finally, the perceived difficulty did not correlate with either the hemodynamic activity or behavioral performance (all *ps* > 0.05).

#### 3.4.2. RNG

The correlational analyses showed a significant negative correlation between the adjacency score and the AUC value of [O_2_Hb] in the left VLPFC (*r* = −0.53; *p* = 0.034) during the RNG1. No relationship was observed between the behavioral data and [O_2_Hb] changes in the RNG2 and RNG3 conditions. These results indicated that a low adjacency score (a better cognitive performance) was associated with a greater activation of the left VLPFC only in the RNG1. Finally, the perceived difficulty did not correlate with either the hemodynamic activity or behavioral performances (all *ps* > 0.05).

## 4. Discussion

The first aim of this study was to examine the evolution of the behavioral performance, subjectively perceived difficulty, and hemodynamic activity in the PFC during the execution of two different cognitive tasks as a function of the cognitive load. The second aim was to explore the relationships between these different parameters. Overall, the main findings showed the general and specific effects of the cognitive load on the subjectively perceived difficulty, cognitive performance, and hemodynamic activity in the PFC for both tasks. However, the relationship patterns between the cognitive performance and hemodynamic activity were less clear and differentiated as a function of the cognitive task. Finally, we observed no functional relationship between the subjectively perceived difficulty and hemodynamic activity.

Regarding the behavioral data, the results showed that during the n-back task, the increase in the cognitive load was accompanied by a decline in the behavioral performance. These findings are consistent with the available literature that showed the effect of the cognitive load on the RT and accuracy measures [7,8,37]. The deterioration of the behavioral performance during the two-back and three-back tasks could be explained by the fact that the amount of information to be updated becomes important during these conditions, and the 3000 ms allotted to the participants to respond did not allow for this update operation to be fully realized. The effect of the cognitive load on behavioral performance (the adjacency score and success rate) was also significant during the RNG task. Indeed, in the RNG3 condition, we observed a greater adjacency score (29.91%) and a lower success rate (0.98) than in the other conditions. These results revealed the reduced inhibition abilities among young adults when the RNG task becomes difficult to perform. The results obtained during the present RNG task are broadly consistent with those of Albinet and collaborators [53] who conducted a study with young adults (mean age = 22 years) using a dual-task procedure in their control condition. Jahanshahi and collaborators also showed that the randomness performance decreased significantly when the cognitive load of the task increased by manipulating the frequency, especially at 1 and 2 Hz [54]. Our results also corroborate those of Towse and Valentine [41] who, manipulating the cognitive load as we did in our study, showed an increase in the adjacency score (from 24.7 to 31.5%). Like these authors, we believe that the difficulty of synchronizing the responses with the generation frequency would explain the reduced inhibitory performance during the RNG3 compared to the RNG1 and RNG2 conditions due to the high cognitive load during the production of habitual or stereotyped responses. Finally, when comparing the behavioral performance of the two tasks (n-back and RNG), even if the metrics are different, an increased cognitive load produced a rather linear decrease in the performance during the n-back task (performance for one-back < two-back < three-back), whereas it was not continuous and linear for the RNG task (performance for RNG1 = RNG2 < RNG3). We will come back to these results later in this section.

The participants’ perceived difficulty Increased gradually as a function of the cognitive load in both tasks. This concurrent evolution of the objective task difficulty and subjectively perceived difficulty is in line with the literature on the mental workload [55,56]. Additionally, the participants perceived the n-back task as significantly more difficult than the RNG task starting from the second cognitive load. This result is important because none of the previous studies have specifically evaluated these self-reports to discriminate the cognitive load of these two tasks subjectively. In the present study, we manipulated the task difficulty so that increasing the amount of information to be updated in the working memory was experienced as more difficult and effortful than increasing the number of information to be inhibited. Future research should verify and expand this finding. However, globally, the behavioral and subjectively perceived difficulty results are concordant with the past literature. They also support our hypotheses and reveal that we correctly manipulated the cognitive load in the present study.

Two issues must be outlined concerning the prefrontal hemodynamic activity measured by fNIRS. First, variations in [O_2_Hb] and [HHb] were globally concordant and in the predicted directions (see Figure 1); however, as often in the fNIRS literature, [O_2_Hb] was more sensitive to experimental manipulations than [HHb], and thus the discussion focuses on the variations of this chromophore. Second, we expressed the relative concentration for each cognitive load as the difference from the reference control task, regardless of the chromophore. Thus, increasing or decreasing the relative concentrations should be reflected in the local brain activity compared to a condition not involving the putative executive process. One important finding is that the n-back and the RNG tasks did not differ significantly in the overall PFC activation (see Section 3.3). Differences emerged as a function of the cognitive load and brain areas. 

Consistent with previous studies, we found a bilateral increase in [O_2_Hb] in the PFC (the DLPFC and VLPFC) during the n-back task [7,14]. Although the difference between the two-back and three-back conditions was insignificant, this increase in [O_2_Hb] showed a significant linear trend as a function of the cognitive load [8,20], and the overall activation was significantly more important in the VLPFC than in the DLPC [18]. This strong VLPFC activity, particularly as a function of the cognitive load (see Figure 4), could be explained by the involvement of the VLPFC in several cognitive processes (selection; comparison and judgment of the stimuli held in the short-term and long-term memory; specification of retrieval cues) essential to the realization of the n-back task [13]. Alternatively, the fact that the increase in the hemodynamic activity was only significant when comparing the two-back and three-back conditions to the one-back condition may reflect the use of different processing strategies, as suggested by Sandrini and collaborators [57]. Concerning the RNG task, the results also showed the effect of the cognitive load on the activation of the PFC (the DLPFC and VLPFC). Like the n-back task, the increase in [O_2_Hb] showed a significant linear trend, but the differences between each condition were insignificant. Thus, compared to the n-back task, the evolution of the PFC brain activity followed the evolution of the cognitive load more parametrically. Following the work of Hoshi and collaborators, a bilateral increase in the hemodynamic activity in the DLPFC and VLPFC was observed during the RNG task [18]. However, contrary to the same authors, our results showed a significantly greater hemodynamic activity in the VLPFC than in the DLPFC. The methodological differences between the two studies could explain this result. In our study, one number was generated every 1.5 s (0.66 Hz), whereas in Hoshi and collaborators’ study [18], one number was generated every 1 s (1 Hz). Moreover, our study used three cognitive loads that required the participants not only to inhibit automatic counting or response schemata but also to avoid certain numbers. The handling of the cognitive load in our study could have induced more mental workload, as opposed to the work by Hoshi and collaborators, which could explain the strong hemodynamic activity observed in the VLPFC beyond the one in the DLPFC. 

Globally, the results regarding the hemodynamic activity of the two executive tasks in the present study suggest that young adults activate their VLPFC more than their DLPFC during the performance of both tasks. This was particularly true for the n-back task, whereas for the RNG task, the activation was more diffuse over the VLPFC and DLPFC. These results confirmed our hypotheses and previous work using other brain imaging techniques, such as fMRI [5] and PET [12]. Of course, these findings are restricted to a limited number of brain areas and should be verified and extended using additional fNIRS channels.

The second objective of the present study was to examine whether the variations in the PFC activity were related more to the cognitive performance or subjectively perceived difficulty. The findings did not support a functional relationship of the perceived subjective difficulty with cognitive performance and the PFC brain activity. In contrast, some relationships specific to some experimental conditions and brain areas emerged between the cognitive performance and PFC activity. During the one-back and three-back tasks, a better cognitive performance was significantly related to a lower activation in the right DLPFC. This can be in line with a previous study involving brain stimulation (transcranial direct current stimulation), that suggests a performance modulation when stimulating the right DLPFC in a task primarily involving a left-lateralized network [58]. Our findings imply that even though the activation during the n-back task was higher in the VLPFC, the improved behavioral performance during the one-back and three-back conditions was functionally related to a lower activation of the right DLPFC. This result might suggest that a redistribution of the blood flow from the DLPFC to the VLPFC would be accompanied by improved the updating performance of the working memory. This explanatory hypothesis should be explored in future studies. During the RNG1, a higher inhibition performance (a low adjacency score) was associated with a greater activation in the left VLPFC, suggesting that a better cognitive performance was functionally associated with a greater activation of the left VLPFC during this task. This result is consistent with that of Shinba and collaborators, who showed in their control subjects that a skillful performance on the RNG task (a low null score and RNG index) increases the activation of the frontal cortex [42]. However, Hoshi and collaborators did not observe a correlation between the activated areas (VLPFC & DLPFC) and the task performance [18]. Overall, when comparing the relationships between the cognitive performance and the PFC hemodynamic activity, we found negative correlations between the n-back task for two conditions (one-back and three-back) within the right DLPFC, whereas for the RNG task, a positive correlation emerged for only one condition (RNG1) within the left VLPFC.

Moreover, concerning the n-back task, the cognitive performance decrease and the PFC activity increase showed a linear trend as a function of the cognitive load. Conversely, for the RNG task, the cognitive performance decrease was not linear, whereas the PFC activity increase followed a linear trend as a function of the cognitive load. Hence, these different measures did not necessarily vary in the same way, and when they covaried, they were not necessarily functionally related. As mentioned in Section 1, controversies still exist concerning the interpretation of an increased PFC activation as a function of the cognitive load; this increasing activity is not always compensatory, that is, being related to a better cognitive performance. Future studies should extensively examine these relationships between the behavioral, subjective, and brain imaging measures and include more brain areas to further these results. 

The present study had some limitations. First, our experimental group contained predominantly males. A large number of males in our sample suggests that the findings observed in the present study would apply more to the male population. Indeed, as shown by Li and collaborators [14], the hemodynamics patterns in the PFC during the n-back task performance seem gender-specific, and our findings on [O_2_Hb] are similar to those observed by these authors in men. Thus, it should be interesting to test the effect of gender in future works using the same experimental protocol. Second, by using only eight channels on the forehead, we did not cover the entire DLPFC and VLPFC, and we could not explore all the brain areas involved in the two tasks used in our study. Other brain regions, such as the superior temporal and parietal cortex, involved in the RNG task [12] and the n-back task [13], should be investigated to overcome this limit. Third, we did not control for either the superficial skin blood flow or systemic effects in our fNIRS signals. Recent methodological articles underline the importance of controlling for these extra cortical contributions to fNIRS signals to optimize the interpretation of the hemodynamic response [59,60,61]. However, as the present study obtained the activation patterns by subtracting the activation of a control condition from each experimental condition, and because the different cognitive loads were counterbalanced, any overall systemic response should have been neutralized. Finally, the sample size for the present study was quite small. Although this sample size is similar or even greater than the one of the previous studies using a similar protocol [7,8,16,17,18,19,20], our study may lack statistical power, and future studies should use a larger sample size to validate the robustness and generalizability of the present results.

## 5. Conclusions

The present study, which investigated the PFC hemodynamics by fNIRS while performing an n-back task and an RNG task with several cognitive loads, yielded the following main findings. Regardless of the executive task, we observed (1) a significant effect of the cognitive load on the behavioral performance, subjectively perceived difficulty, and activation of bilateral PFC; (2) an overall greater activation in the VLPFC compared to DLPFC; and (3) an insignificant relationship between the subjectively perceived difficulty and PFC hemodynamics. Beyond these general outcomes, our study also showed some specificities when comparing the results of the two experimental tasks. First, the participants perceived the n-back task as subjectively more difficult than the RNG task for the two most challenging cognitive loads. However, the patterns of the PFC activity did not reflect these differences. Next, the cognitive performance during the two conditions of the n-back task was related to a lower right DLPFC activation, whereas the cognitive performance during one condition of the RNG task was related to a greater left VLPFC activation. These results highlighted some commonalities but also specificities in the relationship between the cognitive performance and the PFC brain networks as a function of the cognitive load when using two well-known tasks assessing the updating and inhibition of the working memory. They confirm that fNIRS can be used to detect and characterize the PFC activation during the executive tasks varying in cognitive load, but we call for future studies to examine the relationships between the objective and subjective workload and the brain activity in more detail. In addition, future studies should explore more deeply, as suggested by Levy and Wagner [62], the involvement of the subparts (the posterior, mid, and anterior) of the VLPFC that appear to be the most activated cortical regions during the completion of n-back and RNG tasks.

## Figures and Tables

**Table 1:** Effect of cognitive load on behavioral performances.

		Control	Cognitive Load 1	Cognitive Load 2	Cognitive Load 3
n-back	A’ (accuracy)	0.99 ± 0.01	0.98 ± 0.02 *NS*	0.93 ± 0.06 *†	0.85 ± 0.06 *†‡
	RT (ms)	367 ± 41	404 ± 74 *NS*	517 ± 153 *†	593 ± 186 *†‡
RNG	Success rate	-	1±0	0.99 ± 0.01 *NS*	0.98 ± 0.02 †
	Adjacency (%)	-	25.93 ± 8.08	26.88 ± 8.57 *NS*	29.91 ± 9.15 †‡

n-back: control = 0-back; cognitive load 1 = 1-back; cognitive load 2 = 2-back; cognitive load 3 = 3-back. *NS* indicates not significant difference between 0-back and 1-back; * *p* < 0.05 significant difference between 0-back and others cognitive load; † *p* < 0.05 significant difference between 1-back and others cognitive load; ‡ *p* ≤ 0.05 significant difference between 2-back and 3-back. RNG: cognitive load 1 = RNG1; cognitive load 2 = RNG2; cognitive load 3 = RNG 3. *NS* indicates not significant difference between RNG1 and RNG2; † *p* < 0.05 significant difference between RNG1 and RNG3; ‡ *p* < 0.05 significant difference between RNG2 and RNG3.

## Data Availability

The data presented in this study are available on request from the corresponding author.

## Supplementary Materials

The following supporting information can be downloaded at: https://www.mdpi.com/article/10.3390/brainsci12111462/s1, Figure S1: Mean of each participant Reaction Time (ms), A’score (0–1) and perceived difficulty (0–15) as function of cognitive load during the n-back task; Figure S2: Mean of each individual Adjacency score (%), success rate (0–1) and perceived difficulty (0–15) as a function of cognitive load during the RNG task.
