# Supplementary material for: Load-Dependent Prefrontal Cortex Activation Assessed by Continuous-Wave Near-Infrared Spectroscopy during Two Executive Tasks with Three Cognitive Loads in Young Adults
Source: Brain Sci. 2022 Oct 28;12(11):1462. doi: 10.3390/brainsci12111462 (PMC9688545; doi:10.3390/brainsci12111462)
Supplement: Supplementary file 1 [file brainsci-12-01462-s001.zip › brainsci-1955469-supplementary.pdf]

Supplementary Material

n-back task

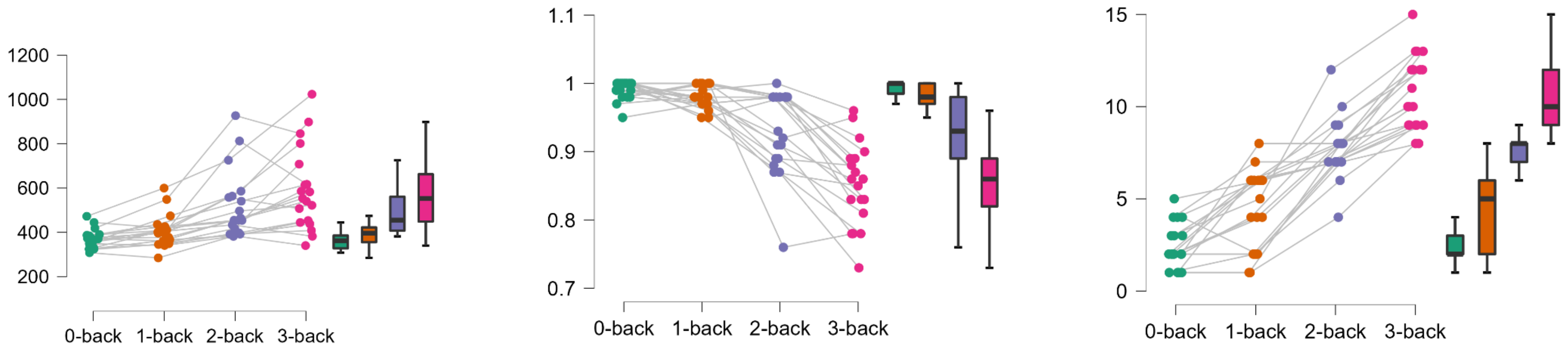

Reaction time

A′ (accuracy)

Perceived difficulty

S.1. Mean of each participant Reaction Time (ms), A′score (0-1) and perceived difficulty (0-15) as function of cognitive load during the n-back task.

Random Number Generation task

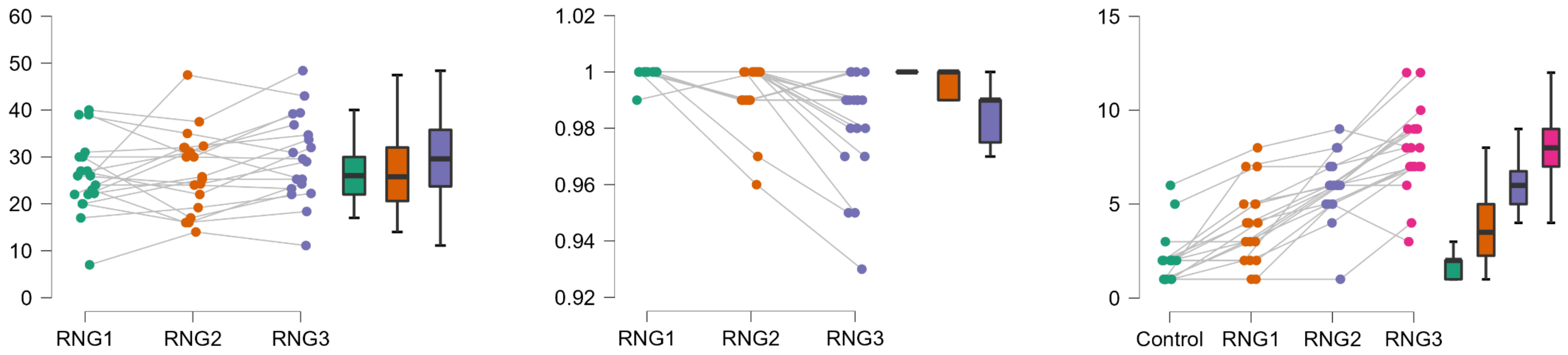

Adjacency

Success rate

Perceived difficulty

S.2. Mean of each individual Adjacency score (%), success rate (0-1) and perceived difficulty (0-15) as a function of cognitive load during the RNG task.
